# Acid-base effects of different crystalloid solutions for ECMO priming: preliminary report

**DOI:** 10.1186/cc14436

**Published:** 2015-03-16

**Authors:** E Scotti, M Ferrari, M Chiodi, F Zadek, I Belloni, L Zazzeron, T Langer, L Gattinoni, P Caironi

**Affiliations:** 1 Fondazione IRCCS Ca' Granda - Ospedale Maggiore Policlinico, Università degli Studi di Milano, Milan, Italy

## Introduction

The induction of ECMO may result in metabolic acidosis [1] due to circuit priming with chloride-rich fluids, and the sudden decrease in plasma strong ion difference (SID). This effect can be attenuated using balanced solutions with a SID equal to the patient's plasma bicarbonate concentration (HCO_3_^-^) [2]. We aimed to compare the effects of a novel balanced solution (SID equal to patients' HCO_3_^-^) with those of commonly employed crystalloids for circuit priming in patients undergoing venovenous ECMO.

## Methods

We randomly assigned patients with acute respiratory failure in need of ECMO to receive either NaCl 0.9% (NS, SID = 0), Ringer lactate (RL, SID = 28), or a novel balanced solution (Solution X, SID equal to the patient's HCO_3_^-^) for circuit priming solution. Arterial blood gases and laboratory parameters were collected at 0, 5, 30, 60, 90, and 120 minutes after pump start. SID, base excess (BE) and total weak acids (Atot) were calculated.

## Results

We enrolled 20 patients (23 priming procedures - RL, *n *= 8; NS, *n *= 8; Solution X, *n *= 7). ECMO was initiated for ARDS (45%), bridge to lung transplant (25%), acute graft failure after transplant (15%), and acute on chronic respiratory failure (15%). Average priming volume was 10 ± 5 ml/kg; patients' baseline HCO_3 _was 28 ± 6 mEq/l. During the first 2 hours after ECMO initiation, arterial pH raised similarly in all groups (*P *= 0.39) due to CO_2_ removal. In contrast, BE decreased starting after 5 minutes in both the NS and RL groups (BE variation, -2.2 ± 1.7 and -1.9 ± 1.3 mEq/l, *P *< 0.001 vs. baseline; *P *= 0.04 for interaction, two-way ANOVA, 2-hour period). No BE changes were observed in the Solution × group (0.3 ± 0.8 mEq/l). In the NS group, BE reduction was associated with a reduction in SID (from 39 ± 8 to 34 ± 6 mEq/l at 5 minutes, *P *= 0.008), entirely due to an increase in Cl (103 ± 7 vs. 108 ± 6 mEq/l, *P *= 0.001). In the RL group, BE and SID reductions (40 ± 8 vs. 36 ± 8 mEq/l, *P *= 0.008) were associated with an increase in both Cl (105 ± 7 vs. 107 ± 7 mEq/l, *P *= 0.01) and lactate (1.4 ± 0.6 vs. 2.2 ± 1.0 mEq/l, *P *= 0.008). No changes were observed in other electrolyte concentrations. Dilution did not differ between groups (*P *= 0.25 for Atot variation). The acidifying effect of NS and RL was amplified in patients with higher baseline HCO_3_^-^.

## Conclusion

As compared with NS and RL, the use of a novel balanced solution with a SID equal to the patient HCO_3_^-^ level for ECMO priming uniquely avoids the addition of metabolic acidosis to patients with uncompensated hypercapnia.
